# Efficient Screening in Obstructive Sleep Apnea Using Sequential Machine Learning Models, Questionnaires, and Pulse Oximetry Signals: Mixed Methods Study

**DOI:** 10.2196/51615

**Published:** 2024-12-19

**Authors:** Nai-Yu Kuo, Hsin-Jung Tsai, Shih-Jen Tsai, Albert C Yang

**Affiliations:** 1 Sleep Medicine Center Taipei Veterans General Hospital Taipei Taiwan; 2 Institute of Brain Science National YangMing Chiao Tung University Taipei Taiwan; 3 Department of Psychiatry Taipei Veterans General Hospital Taipei Taiwan; 4 Department of Medical Research Taipei Veterans General Hospital Taipei Taiwan; 5 Digital Medicine and Smart Healthcare Research Center National Yang Ming Chiao Tung University Taipei Taiwan

**Keywords:** sleep apnea, machine learning, questionnaire, oxygen saturation, polysomnography, screening, sleep disorder, insomnia, utilization, dataset, training, diagnostic

## Abstract

**Background:**

Obstructive sleep apnea (OSA) is a prevalent sleep disorder characterized by frequent pauses or shallow breathing during sleep. Polysomnography, the gold standard for OSA assessment, is time consuming and labor intensive, thus limiting diagnostic efficiency.

**Objective:**

This study aims to develop 2 sequential machine learning models to efficiently screen and differentiate OSA.

**Methods:**

We used 2 datasets comprising 8444 cases from the Sleep Heart Health Study (SHHS) and 1229 cases from Taipei Veterans General Hospital (TVGH). The Questionnaire Model (Model-Questionnaire) was designed to distinguish OSA from primary insomnia using demographic information and Pittsburgh Sleep Quality Index questionnaires, while the Saturation Model (Model-Saturation) categorized OSA severity based on multiple blood oxygen saturation parameters. The performance of the sequential machine learning models in screening and assessing the severity of OSA was evaluated using an independent test set derived from TVGH.

**Results:**

The Model-Questionnaire achieved an *F*_1_-score of 0.86, incorporating demographic data and the Pittsburgh Sleep Quality Index. Model-Saturation training by the SHHS dataset displayed an *F*_1_-score of 0.82 when using the power spectrum of blood oxygen saturation signals and reached the highest *F*_1_-score of 0.85 when considering all saturation-related parameters. Model-saturation training by the TVGH dataset displayed an *F*_1_-score of 0.82. The independent test set showed stable results for Model-Questionnaire and Model-Saturation training by the TVGH dataset, but with a slightly decreased *F*_1_-score (0.78) in Model-Saturation training by the SHHS dataset. Despite reduced model accuracy across different datasets, precision remained at 0.89 for screening moderate to severe OSA.

**Conclusions:**

Although a composite model using multiple saturation parameters exhibits higher accuracy, optimizing this model by identifying key factors is essential. Both models demonstrated adequate at-home screening capabilities for sleep disorders, particularly for patients unsuitable for in-laboratory sleep studies.

## Introduction

Obstructive sleep apnea (OSA) is a prevalent sleep-related breathing disorder resulting from reduced muscle tension and collapsed soft tissue, causing airway blockage or limited airflow. This leads to fragmented sleep [1], nocturnal hypoxia [2], intrathoracic pressure fluctuations [3], daytime sleepiness [4], cognitive decline [5,6], and increased cardiovascular and metabolic disease risk [7-9]. OSA negatively impacts patients’ long-term health, and quality of life, and increases medical and societal burdens [10]. An 11-year analysis by the US Veterans Health Administration identified sleep apnea and insomnia as the most common sleep disorders, at 47% and 26%, respectively [11]. Nearly 1 billion people worldwide experience from sleep apnea, with 430 million experiencing moderate to severe cases [12]. Prevalence increases with age, male gender, and higher BMI [13].

Polysomnography is the gold standard for diagnosing sleep apnea, requiring overnight monitoring in a sleep center with sensors detecting physiological changes. Respiratory signals, such as airflow, respiratory effort, snoring, and blood oxygen saturation (SpO_2_), are analyzed by sleep technicians to calculate the Apnea-Hypopnea Index (AHI), which classifies apnea severity. Moderate to severe cases (ie, AHI values ≥ 15) require further treatment and management.

The prevalence of sleep apnea is quite high, yet low diagnosis rates persist. In a telephone survey of 4011 Taiwanese people, 51.9% reported snoring symptoms, and 2.6% were observed to have sleep apnea [14], indicating many potential patients may still be undiagnosed and untreated compared with other Asian regions [15]. Low diagnosis rates may be due to polysomnography limitations. Unfamiliar environments and equipment can cause discomfort and affect sleep patterns, potentially leading to misdiagnosis. The labor-intensive process requires trained technicians to analyze only three patients per night, causing longer waiting times. Manual analysis introduces variability in interpretation, with past research showing significant discrepancies in AHI values [16]. COVID-19 has further complicated hospital visits for testing, reducing confidence in diagnosis and increasing health care system burdens.

Recent studies aim to provide comfortable and convenient sleep apnea screening options, using subjective methods like questionnaires (eg, STOP-Bang, an acronym for Snoring, Tiredness, Observed apnea, high Blood Pressure, BMI, age, neck circumference, and gender) or objective methods involving wearable devices. The STOP-Bang questionnaire has high sensitivity but low specificity [17], potentially misclassifying patients. Objective methods analyze signals from wearables, such as electrocardiogram signals combined with cardiopulmonary coupling and cyclic variation of heart rate, achieving 89% sensitivity and 79% specificity [18]. Pulse oximeters are popular in research due to their simplicity and affordability [19].

The primary objective of this study is therefore to create a patient-friendly, clinically applicable tool for detecting OSA using machine learning techniques. This tool will combine subjective clinical questionnaires with objective blood oxygen concentration signals. In total, 2 distinct models have been developed: the Questionnaire Model (Model-Questionnaire) and the Saturation Model (Model-Saturation).

The Model-Questionnaire collects relevant questionnaires from a database, which includes demographic information and the Pittsburgh Sleep Quality Index (PSQI). Machine learning is then performed on these selected items. The Model-Saturation, on the other hand, uses SpO_2_ signals. This model involves preprocessing to eliminate noise interference and uses various methods for feature extraction, such as time-domain analysis, frequency-domain analysis, nonlinear analysis, and estimation of oxygen desaturation index.

In this study, we used 2 extensive polysomnography datasets, the Sleep Heart Health Study (SHHS) [20,21] and polysomnography records from the Sleep Medicine Center of Taipei Veterans General Hospital (TVGH), for the training, testing, and validation of both models. We justified using datasets from different geographic areas to enhance the robustness and generalizability of their machine learning models across diverse populations, addressing potential biases and improving the study’s external validity. Furthermore, we will assess these models using independent test data derived from TVGH to confirm the combined process’s effectiveness and applicability.

This approach presents several advantages. For example, self-administered questionnaires can be implemented across various medical settings, increasing awareness among high-risk patients who might be unaware of their sleep apnea. In addition, pulse oximeters offer a noninvasive, cost-effective, and convenient solution for long-term monitoring, serving as an alternative to standard polysomnography. The unique aspect of this study lies in the integration of these methods for preliminary screening and triaging patients with OSA, ultimately promoting more efficient and accessible diagnosis and treatment options.

## Methods

### Sleep Data

We used 2 primary databases for this purpose: the clinical sleep data from the Sleep Medicine Center at TVGH, and the SHHS from the National Sleep Research Resource in the United States.

The Sleep Medicine Center’s clinical sleep database, operated by the Department of Psychiatry at TVGH, contains clinical questionnaires and full-night standard polysomnography data for 4132 patients collected between December 1, 2012, and December 31, 2019. These questionnaires included clinical information, the PSQI, the Insomnia Severity Index, the Epworth Sleepiness Scale, and the Beck Depression Inventory-II. Sleep studies were conducted at the Sleep Medicine Center of Taipei Veterans General Hospital using Embla N7000 equipment (Natus Medical Inc), and data analysis was performed using RemLogic Version 3.4.1 software (Natus Medical Inc).

The SHHS is a multicenter cohort study designed to test whether sleep-related breathing is associated with an increased risk of cardiovascular disease. The study was conducted using the home-based type II polysomnography device between November 1, 1995, and January 31, 1998, and involved 6441 men and women aged 40 years and above for the initial evaluation and sleep study. Between January 2001 and June 2003, 3295 participants underwent a second polysomnography. The SHHS database provides 8444 records of home-based polysomnography data with rigorous scoring. For the purposes of this study, we used the provided 8444 records and extracted the SpO_2_ signals for Model-Saturation training.

### Data Preparation and Preprocessing

**Figure 1:**
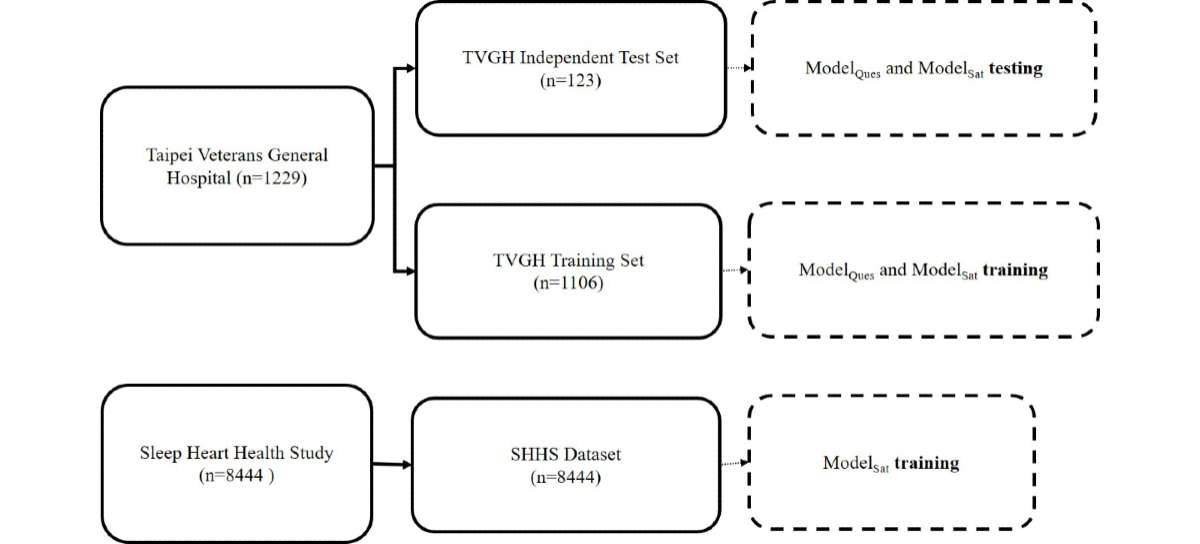
Structure of Polysomnographic Data from Sleep Heart Health Study and Taipei Veterans Hospital. ModelQues: Model-Questionnaire; ModelSat: Model-Saturation. SHHS: Sleep Heart Health Study; TVGH: Taipei Veterans General Hospital.

To develop 2 models for predicting the presence and severity of OSA, the data will be divided into three groups: (1) the TVGH training set and the SHHS dataset, (2) both used for constructing the models, and (3) a TVGH independent test set for evaluating the models’ performance, as illustrated in Figure 1.

Initially, 1229 patients were selected from the TVGH database, comprising 822 patients diagnosed with OSA and 407 patients with primary insomnia through polysomnography. These patients were split into the TVGH Model-Questionnaire training set and the TVGH independent test set at a 9:1 ratio. The cases were categorized into 3 groups based on the severity of their condition: no sleep apnea, mild sleep apnea, and moderate-to-severe sleep apnea. To ensure a more uniform distribution of AHI across both datasets, participants in each group were sorted by age and AHI. Every 10th participant was chosen in sequence, resulting in 123 participants for the TVGH independent test set and 1106 participants for the TVGH Model-Questionnaire training set.

All 8444 sleep study records in the SHHS database served as the SHHS Model-Saturation training set. In addition, the polysomnography data obtained from these 1106 participants at TVGH also served as a training dataset for the Model-Saturation, which was trained separately from the SHHS database. SpO_2_ signals were obtained from both the SHHS and TVGH databases. However, due to the different equipment used, the sampling frequency of SpO_2_ signals varied between the 2 databases, 1 Hz in the SHHS database and 10 Hz in the TVGH database. To achieve signal consistency, the blood oxygen concentration signal in the TVGH database was down-sampled to 1 Hz. After standardizing the sampling frequency, SpO_2_ signals underwent a three-step preprocessing procedure, which involved (1) excluding data points with blood oxygen concentrations below 50; (2) eliminating data exhibiting abrupt drops in blood oxygen concentration signals exceeding 4% within 1 second; and (3) excluding noncontinuous blood oxygen concentration signals for over 1 minute, to eliminate noise arising from instrument disconnection or participant movement during monitoring.

### Feature Extraction

#### Model-Questionnaire

The Model-Questionnaire used questionnaires as the screening process for determining the presence of sleep apnea. Clinical data collected by the questionnaires and the PSQI served as feature values. Clinical data included gender, age, BMI, and the patient’s history of hypertension, diabetes, and cardiovascular disease, resulting in a total of 6 items.

The PSQI is a retrospective questionnaire that prompts participants to recall their sleep patterns over the past month. It consists of 18 self-reported questions. Once completed, a specific calculation method is used to derive 7 subscores representing subjective sleep quality, sleep latency, sleep duration, sleep efficiency, sleep disturbance, medication use, and daytime dysfunction. The subscores are then summed to produce the final PSQI score. This study used 18 self-reported questions, 7 subscores, and the final PSQI score, a total of 26 items, as feature values.

#### Model-Saturation

The Model-Saturation used SpO_2_ signals to classify patients’ sleep apnea severity. After preprocessing the blood oxygen concentration signal, four methods were used to extract signal features:

##### Saturation Distribution

Sleep-disordered breathing indirectly influences blood oxygen concentration changes. The average blood oxygen concentration distribution graph, plotted after grouping participants with varying apnea severities, indicates changes in blood oxygen concentration distribution based on severity. This study calculated the mean, median, maximum, minimum, kurtosis, variance, and skewness of SpO_2_ signal from the collected overnight signals to describe the blood oxygen concentration distribution and use these 7 parameters as feature values.

##### Power Spectral Density

Sleep-disordered breathing induces fluctuations in blood oxygen concentration. Therefore, frequency analysis can be used to assess the oxygen desaturation index. By analyzing the power spectral density of the signal from the time domain to the frequency domain, the proportion of different frequencies can be obtained. This study will use 100 feature values acquired at intervals of 0.001 Hz from 0 to 0.1 Hz.

##### Multiscale Entropy

This study also attempted to use multiscale entropy (MSE) to detect changes in blood oxygen concentration. As a nonlinear analysis method, MSE observes the complexity of the signal at different time scales by using data points of varying lengths and averaging their values [22]. It then calculates the sample entropy [23] at different scales. This study used 20 scales and adopted these 20 items as feature values.

##### Approximation of Oxygen Desaturation Index

The oxygen desaturation index is defined as the average number of times that blood oxygen concentration drops by more than 4% per hour during an entire night’s sleep. However, this study only used a single signal and cannot determine the patient’s sleep and wake times. Therefore, the total number of times blood oxygen concentration decreases by 4% is divided by the total recording time to obtain an approximate value of the oxygen desaturation index.

In summary, the Model-Saturation adopts a total of 128 feature values, including 7 features from saturation distribution, 100 features from Power spectral density (PSD), 20 features from MSE, and 1 feature from the approximation of oxygen desaturation index (ODI).

### Ground Truth Labels for Machine Learning Models

#### Model-Questionnaire

This study used the database from TVGH for training and testing the Model-Questionnaire. Patients with an AHI ≥ 5 after undergoing overnight polysomnography were labeled as having OSA. In contrast, those diagnosed with primary insomnia by a physician, without any other sleep disorders detected during polysomnography, were labeled as having primary insomnia. The Model-Questionnaire used 6 features from clinical data and 26 features from the PSQI, targeting OSA and primary insomnia as the ground truth values obtained from polysomnography results.

#### Model-Saturation

The Model-Saturation used preprocessed and feature-extracted SpO_2_ signals to further categorize the severity of OSA in patients identified by the Model-Questionnaire. In this study, the AHI defined OSA severity. Patients with an AHI of <5 were labeled as without sleep apnea, those with an AHI of ≥5 but <15 were labeled as having mild sleep apnea, and those with an AHI of ≥15 were labeled as having moderate to severe sleep apnea. To ensure consistency among different databases, this study adopted the criteria published by the American Academy of Sleep Medicine (AASM) in 2012 to calculate the AHI. Apnea was defined as a reduction in airflow by ≥90% lasting for at least 10 seconds, while hypopnea was defined as a reduction in airflow by ≥30% lasting for at least 10 seconds accompanied by ≥4% oxygen desaturation. The total number of apnea and hypopnea events was divided by the total sleep time to obtain the AHI.

### Machine Learning Models

In building the machine learning models, this study used a machine learning training platform (Invent AI by Chunghwa Telecom in Taiwan) to train and establish the Model-Questionnaire and Model-Saturation. TVGH independent test set was used to test the process after creating the 2 models. Three algorithms, including CatBoost, Random Forest, and Extra Trees were used in the AI platform for this study.

During the modeling process, the training sets for both the Model-Questionnaire and the Model-Saturation were divided into 3 sets by a fixed ratio. Specifically, 70% (Model-Questionnaire: 774/1106, Model-Saturation: 5910/8444) was allocated for training, 15% (Model-Questionnaire: 166/1106, Model-Saturation: 1267/8444) for validation, and the remaining 15% (Model-Questionnaire: 166/1106, Model-Saturation: 1267/8444) of the data was designated as the testing set for each model. Synthesized Minority Oversampling Technique method, which is an oversampling technique that artificially increases the data by interpolating among the minority samples, was used to deal with the issue of data imbalance. After the model building was completed, the reserved TVGH independent test set of 123 samples was used to evaluate whether the 2 models could classify OSA well in a sequential manner. First, the questionnaire data in the TVGH independent test set was inputted into the established Model-Questionnaire, resulting in the classification of primary insomnia and OSA. Then, the SpO_2_ signal from cases predicted by Model-Questionnaire to have OSA was used in Model-Saturation, resulting in the final classification into three categories: (1) without sleep apnea, (2) mild sleep apnea, and (3) moderate to severe sleep apnea.

### Statistical Analysis and Model Evaluation

As the data for this study were obtained from 2 different databases and was subsequently divided into 3 datasets for model training and testing, descriptive statistics were used to calculate the mean and SD of age, BMI, and AHI for each dataset. The number and proportion of cases by gender and severity of OSA were also calculated.

To evaluate the machine learning models, this study generated a confusion matrix by comparing the predicted values generated by the model with the actual classifications. *F*_1_-score, precision, recall, accuracy, and area under the curve (AUC) were calculated to assess the predictive ability of the classifiers. The independent test dataset was used to validate the established model-questionnaire and model-saturation, and the results were used to generate a confusion matrix. *F*_1_-score, precision, recall, and accuracy were calculated to evaluate the model’s performance.

In this study, we also presented the ranking of feature importance based on the weights in the algorithms. The weights, which indicate the number of times a feature appears across all trees, were retrieved from the model to evaluate the feature importance, thereby yielding the average gain associated with each feature and highlighting how each contributes to the model’s decision-making process.

### Ethical Considerations

This study was approved by the institutional review board of Taipei Veterans General Hospital (approval number 2020-09-008BC) for the TVGH dataset, and approval for the use of data from the National Sleep Research Resource (NSRR R24 HL114473: NHLBI National Sleep Research Resource) was also obtained. Both databases do not contain any personally identifiable information or sensitive individual data, and the researchers were unable to determine the identities of the participants or contact them.

## Results

### Data Characteristics

This study used 2 large databases and divided them into 3 datasets for training and validation of the models. Table 1 displays the demographic data for the 3 datasets. The TVGH training set and independent testing datasets were both from the TVGH database. The SHHS dataset had slight differences in gender, age, and AHI distribution compared with the other 2 datasets. This was because the SHHS database included participants over the age of 40, while the TVGH database included participants over the age of 18 years who underwent sleep studies for sleep disorders. These differences in data collection purpose can affect the results but also provide insight into whether using a Model-Saturation trained from the SHHS dataset can predict well for Taiwanese data.

As shown in Table 1, both databases had data imbalance issues. The proportions of patients with primary insomnia and those with OSA in the TVGH training set were 33.1% (366/1106) and 66.9% (740/1106), respectively, while the SHHS dataset included 46.2% (3904/8444) of no sleep apnea, 31.2% (2630/8444) of mild sleep apnea, and 22.6% (1910/8444) of moderate-to-severe sleep apnea. Since data imbalance can affect the model’s prediction, we employed the Synthesized Minority Oversampling Technique method to balance the data.

**Table 1:** Demographic data of 3 datasets.

		SHHS^a^ dataset (n=8444)	TVGH^b^ training set (n=1106)	TVGH independent test det (n=123)
**Sex, n** **(%)**				
	Male	3986 (47.2)	651 (58.9)	69 (56.1)
	Female	4458 (52.8)	455 (41.1)	54 (43.9)
Age (years), mean (SD)		64.55 (11.17)	52.35 (15.61)	51.80 (15.16)
BMI (kg/m^2^), mean (SD)		28.21 (5.79)	25.04 (4.82)	25.20 (4.56)
AHI^c^ (events/hour), mean (SD)		10.67 (13.78)	17.43 (21.08)	16.84 (19.88)
**Severity of Sleep Apnea, n** **(%)**				
	Normal (AHI<5)	3904 (46.2)	366 (33.1)	41 (33.3)
	Mild (5≦AHI<15)	2630 (31.2)	325 (29.4)	36 (29.3)
	Moderate (15≦AHI<30)	1218 (14.4)	194 (17.5)	22 (17.9)
	Severe (30≦AHI)	692 (8.2)	221 (20.0)	24 (19.5)

^a^SHHS: Sleep Heart Health Study.

^b^TVGH: Taipei Veterans General Hospital.

^c^AHI: Apnea-Hypopnea Index.

### Model-Questionnaire

The training results of Model-Questionnaire for different algorithms and feature selection are presented in Table 2. Using all features for model training resulted in the same accuracy for all three algorithms. However, CatBoost had a better *F*_1_-score of 0.85 compared with the other 2 algorithms. The feature importance ranking showed that BMI, age, self-reported snoring, gender, and hypertension history were the most frequently occurring 5 features. Reducing the features to only these 5 and training the model again resulted in a slightly improved *F*_1_-score and accuracy compared with using all features. The best algorithm for this reduced feature model was ExtraTrees, which may be related to the preselection of features.

**Table 2:** Results of Model-Questionnaire.

Model name		*F*_1_-score	Precision	Recall			Accuracy (%)		AUC^a^	Feature importance									
										High								Low	
**All features**																			
	CatBoost	0.85^b^	0.86		0.86	86.49%		0.84			BMI		Age		PSQI^c^		Gender		PSQI^d^
	ExtraTrees	0.85	0.87		0.86	86.49%		0.85			Age		PSQI^a^		Gender		BMI		HBP^e^
	Random Forest	0.84	0.88		0.86	86.49%		0.86			BMI		Age		PSQI^c^		Gender		HBP
**5 features**																			
	CatBoost	0.86	0.86		0.86	86.49%		0.84			Age		BMI		PSQI^c^		Gender		HBP
	ExtraTrees	0.86^b^	0.87		0.87	87.39%		0.86			Age		PSQI^a^		BMI		Gender		HBP
	Random Forest	0.86	0.87		0.87	87.39%		0.86			BMI		Age		PSQI^c^		Gender		HBP

^a^AUC: Area Under Curve.

^b^The machine learning model with the highest *F*_1_-score.

^c^PSQI: PSQI question 5.5 (During the past month, how often have you had trouble sleeping because you cough or snore loudly?).

^d^PSQI: PSQI question 5.4 (During the past month, how often have you had trouble sleeping because you cannot breathe comfortably?).

^e^HBP: high blood pressure.

### Model-Saturation

#### Model Training Using the Sleep Heart Health Study Dataset

The training results of Model-Saturation for different algorithms and feature selection are presented in Table 3. Using all features for model training, CatBoost had the best *F*_1_-score of 0.85 and an accuracy of 84.5% among the 3 algorithms. The feature importance ranking showed that the estimated oxygen desaturation index, MSE, and spectral density analysis were all important features. However, after removing the SpO_2_ distribution–related parameters, the model performance was not as good as using all features. Using single feature analysis for model training (Table 4), the model using spectral density analysis with Random Forest had the best prediction performance, with an *F*_1_-score of 0.82 and an accuracy of 82%.

**Table 3:** Results of Model-Saturation by Sleep Heart Health Study dataset.

Model name		*F*_1_-score	Precision	Recall	Accuracy (%)	AUC^a^	Feature importance				
							High				Low
**All features**											
	CatBoost	0.85^b^	0.85	0.84	84.47%	0.95	ODI^c^	MSE1^d^	PSD^e^ (0.013 Hz)	PSD (0.020 Hz)	PSD (0.017 Hz)
	ExtraTrees	0.81	0.81	0.81	80.55%	0.94	ODI	MSE1	PSD (0.013 Hz)	PSD (0.016 Hz)	PSD (0.021 Hz)
	Random Forest	0.83	0.83	0.83	83.19%	0.95	PSD (0.020 Hz)	PSD (0.019 Hz)	PSD (0.022 Hz)	PSD (0.021 Hz)	PSD (0.017 Hz)
**ODI+MSE+PSD**											
	CatBoost	0.84^b^	0.84	0.84	83.87%	0.95	ODI4	MSE1	PSD (0.022 Hz)	PSD (0.017 Hz)	PSD (0.019 Hz)
	ExtraTrees	0.82	0.82	0.82	81.57%	0.94	MSE1	ODI4	PSD (0.019 Hz)	MSE3	PSD (0.020 Hz)
	Random Forest	0.79	0.80	0.79	78.58%	0.93	PSD (0.020 Hz)	PSD (0.019 Hz)	PSD (0.021 Hz)	PSD (0.024 Hz)	PSD (0.017 Hz)

^a^AUC: area under curve.

^b^The machine learning model with the highest *F*_1_-score.

^c^ODI: oxygen desaturation index.

^d^MSE1: multiscale entropy.

^e^PSD: power spectral density.

**Table 4:** Model training results of SpO_2_^a^ signal by single feature analysis

Model name			*F*_1_-score		Precision		Recall		Accuracy, %		Area under curve
**Power spectrum density**											
	CatBoost	0.81		0.81		0.81		80.89		0.94	
	ExtraTrees	0.79		0.80		0.79		79.10		0.93	
	Random Forest	0.82^b^		0.82		0.82		82.00		0.94	
**Multiscale entropy**											
	CatBoost	0.70		0.70		0.70		69.54		0.86	
	ExtraTrees	0.69		0.69		0.69		68.77		0.86	
	Random Forest	0.71^b^		0.72		0.71		71.25		0.87	
**Saturation distribution**											
	CatBoost	0.67^b^		0.67		0.67		67.32		0.84	
	ExtraTrees	0.65		0.66		0.65		65.27		0.83	
	Random Forest	0.66		0.67		0.67		67.06		0.84	
**Approximation of oxygen desaturation index**											
	CatBoost	0.67^b^		0.67		0.66		66.30		0.83	
	ExtraTrees	0.66		0.67		0.66		66.30		0.82	
	Random Forest	0.66		0.67		0.66		66.21		0.83	

^a^SpO_2_: blood oxygen saturation.

^b^The machine learning model with the highest *F*_1_-score.

#### Model Training Using the Taipei Veterans General Hospital Dataset

Table 5 presents the Model-Saturation built for 1106 cases in the TVGH training dataset. When using all features, the CatBoost algorithm achieves the best *F*_1_-score of 0.83 and an accuracy of 0.82. The feature importance, in order, includes ODI, power spectral density, and variance as well as kurtosis of SpO_2_ signal.

**Table 5:** Results of Model-Saturation using the Taipei Veterans General Hospital training set.

Model name	*F*_1_-score	Precision	Recall	Accuracy (%)	AUC^a^	Feature importance				
						High				Low
CatBoost	0.82^b^	0.83	0.82	82.40%	0.94	ODI^c^	PSD^d^ (0.022 Hz)	SpO_2_ Variance	PSD (0.014 Hz)	SpO_2_ Kurtosis
ExtraTrees	0.81	0.83	0.80	80.80%	0.92	ODI	PSD (0.022 Hz)	MSE9	PSD (0.039 Hz)	MSE2
Random Forest	0.82	0.83	0.81	81.60%	0.93	ODI	PSD (0.020 Hz)	SpO_2_ Variance	PSD (0.014 Hz)	PSD (0.025 Hz)

^a^AUC: area under the curve.

^b^The machine learning model with the highest *F*_1_-score.

^c^ODI: Oxygen desaturation index.

^d^PSD: Power spectral density.

### Validation of Models Using TVGH Independent Test Data

The ultimate purpose of this study was to use 2 machine learning models to screen patients who may have OSA for further diagnosis and triage. Therefore, the best Model-Questionnaire and Model-Saturation were selected from the training models for use in the final validation. Based on the results in Table 2, the Extra Trees algorithm using the 5 features had good *F*_1_-score, accuracy, and recall, and was chosen as the final Model-Questionnaire for the screening process. The Model-Saturation using all features with CatBoost was chosen for the second step of OSA triage, as it had the best performance in terms of recall, precision, accuracy, and *F*_1_-score compared with other combinations.

**Figure 2:**
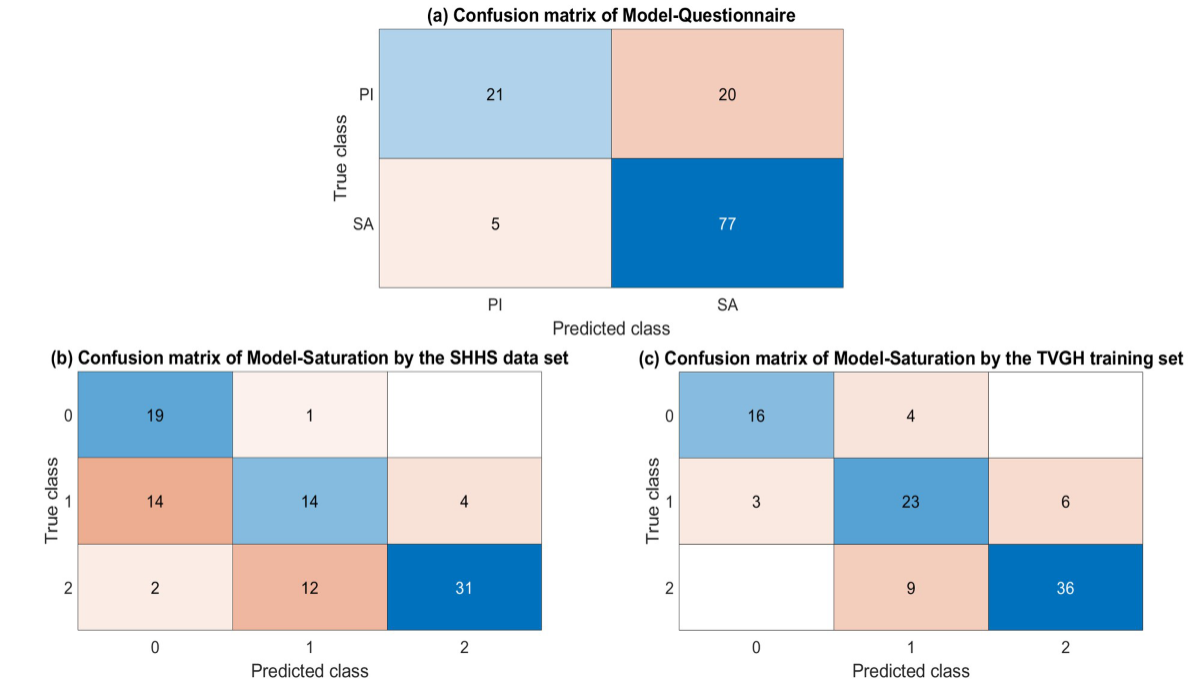
Confusion Matrix of Two Models: (a) Confusion matrix of Model-Questionnaire (b) Confusion matrix of Model-Saturation by the Sleep Heart Health Study data set (c) Confusion matrix of Model-Saturation by the Taipei Veterans General Hospital training set. PI: Primary insomnia; SA: Sleep apnea; 0: Without sleep apnea; 1: Mild sleep apnea; 2: Moderate and severe sleep apnea. SHHS: Sleep Heart Health Study; TVGH: Taipei Veterans General Hospital.

The TVGH independent test dataset contains 123 cases, all of which included questionnaire data and SpO_2_ signal. For the Model-Questionnaire, the results using the independent testing dataset are shown in Table 6. Although the accuracy and precision are lower than those in Table 2, the recall rate is 0.94, indicating that the model has a high sensitivity to the independent testing dataset. The *F*_1_-score is also slightly higher. The confusion matrix in Figure 2a shows that although almost half of the 41 patients with primary insomnia were classified as having sleep apnea, 77 out of 82 patients with true sleep apnea were correctly classified, which suggests the model has high sensitivity despite the lower precision.

For the Model-Saturation, 97 cases classified as having sleep apnea by the Model-Questionnaire were further classified according to the severity of sleep apnea. Table 6 shows that although the accuracy of the Model-Saturation for moderate-to-severe sleep apnea is only 65.98% (64/97), which is significantly lower than the result obtained from the SHHS dataset (as shown in Table 3), this may be due to differences between the databases. However, the confusion matrix (Figure 2b) shows that, among those classified as having moderate-to-severe sleep apnea, all but four patients who actually had mild sleep apnea were correctly classified. The precision also slightly increased to 0.89, indicating that if a patient is classified as having moderate-to-severe sleep apnea by the Model-Saturation, there is a high possibility of having moderate-to-severe sleep apnea. Therefore, this Model-Saturation is considered effective in achieving the initial screening goal of moderate-to-severe sleep apnea even when using databases from different sources.

Finally, when tested on a TVGH-independent test set, the Model-Saturation trained using the TVGH training set showed an improved *F*_1_-score from 0.775 to 0.828 (Table 6) compared with the Model-Saturation trained with the SHHS database. The results were closer to the accuracy obtained from the TVGH training set, and as shown in Figure 2c, the Model-Saturation trained with TVGH data demonstrates better performance in classifying patients with mild and moderate-severe sleep apnea compared with the Model-Saturation trained on the SHHS database.

**Table 6:** Testing results of two models by independent testing dataset.

Model			*F*_1_-score		Precision		Recall		Accuracy, %
**Model-Questionnaire**									
	ExtraTrees	0.8603		0.7938		0.939		79.67	
**Model-Saturation using the** **Sleep Heart Health Study** **dataset**									
	CatBoost	0.775		0.8857		0.6889		65.98	
**Model-Saturation using the** **Taipei Veterans General Hospital** **dataset**									
	CatBoost	0.8276		0.8571		0.8000		77.32	

## Discussion

### Principal Findings

To the best of our knowledge, this study is the first to use a large amount of polysomnography data to establish sequential questionnaire and saturation models as a preliminary screening process for OSA. Previous studies mostly used a combination of questionnaires and blood oxygen signal features for prediction rather than building 2 models separately. For example, Mashaqi et al [24] used the STOP-Bang questionnaire combined with ODI to predict sleep apnea but achieved better classification only for severe cases.

However, if we can use a questionnaire to quickly and easily classify potential patients and then use SpO_2_ signals to classify patients with moderate to severe sleep apnea who need to be diagnosed through polysomnography tests, it can improve the efficiency of sleep examinations and reduce unnecessary medical resource consumption. Therefore, compared with previous studies that used a model combining questionnaire and blood oxygen concentration for prediction, this study constructed a process consisting of 2 machine learning models that are more suitable for clinical applications.

### Model-Questionnaire

The Model-Questionnaire was established using PSQI questionnaire data collected from the TVGH and was mainly used to distinguish between patients with sleep apnea and those with primary insomnia. As these are the 2 most common sleep disorders, the established model can cover a wide range of clinical applications. Based on the results in Table 2, using only the 5 features with the highest importance for the ExtraTrees algorithm, namely age, gender, BMI, snoring status, and history of hypertension, resulted in the highest accuracy for the questionnaire model while also improving model efficiency. These 5 features have been proven in previous studies to be risk factors for sleep apnea and are similar to STOP-Bang questionnaire items [17].

Although the STOP-Bang questionnaire has high sensitivity but low accuracy in detecting sleep apnea, with an AUC of only 0.56 in East Asian populations [25], our Model-Questionnaire achieved an AUC of 0.86, indicating better performance. Furthermore, since the database used for the questionnaire model only included data for sleep apnea and primary insomnia diagnoses, the trained model classified cases into these 2 categories. However, based on the important features for screening sleep apnea exhibited in the Model-Questionnaire, it may be possible to more broadly classify patients as having or not having sleep apnea.

### Model-Saturation

Since the Model-Questionnaire has high sensitivity but low accuracy, it may result in misclassifying patients without sleep apnea. Therefore, it is important to further classify patients using physiological signals, which led to the development of the Model-Saturation in this study. Four different analyses were used to obtain features from the SpO_2_ signal, and sleep apnea was classified into 3 different severity levels.

According to the feature importance in Table 3, the estimated ODI is the most important parameter in the model. Hang et al [26] used a support vector machine algorithm with ODI to predict moderate to severe sleep apnea and achieved an accuracy rate of 87.3%-87.8%. However, the accuracy rate of this study’s model using only ODI as a feature was only 66.3%, which may be due to differences in signal quality and feature analysis methods. The data used in the study by Hang et al [26] were obtained from a single sleep center and SpO_2_ signals were collected through traditional sleep tests. In contrast, this study used the SHHS database, which is a multicenter home sleep test that cannot control for artifacts and other interferences, making it more challenging to estimate ODI from blood oxygen signals. However, this approach is more representative of real-world clinical data collected from patients at home. In addition, the calculation of ODI in this study only considered oxygen desaturation of 4%, while Hang et al. included both 2% and 4% desaturation. This difference in methodology may be worth considering in future studies to improve the results.

In terms of feature importance, sample entropy (ie, entropy obtained in the original time series calculation under a time scale of 1) was found to be the second most important feature after ODI. Previous studies have also used MSE to study pediatric sleep apnea and calculated sample entropy over a range of time scales from 1 to 6. However, the final model only included sample entropy calculated under a time scale of 1 [27]. Based on the results of this study and previous literature, using sample entropy directly calculated from the original signal may be more efficient than using a time-consuming preprocessing method such as MSE.

Table 4 shows that the highest accuracy rate was achieved using spectral density analysis, with an accuracy rate of 82% and an AUC of 0.94. This feature also ranked high in the feature importance analysis in Table 3. Alvarez et al also found that spectral density analysis had better accuracy in classifying sleep apnea compared with other time-domain and nonlinear analysis methods. Among the features obtained through spectral density analysis, the peak amplitude between 0.014 Hz and 0.033 Hz had the highest accuracy rate of 83.1% [28]. In this study, using an interval of 0.001 Hz, the most important frequencies in the top 5 features were found to be between 0.013-0.014 Hz, indicating that the duration of blood oxygen desaturation of around 71.4-76.9 seconds is a discriminating factor for classifying the severity of sleep apnea.

In addition, from Table 5, which presents the Model-Saturation built for the 1106 cases at TVGH, it is evident that the importance of the variance of SpO_2_ signal has significantly increased. In spectral analysis, the feature importance has slightly increased, and the frequency adopted is somewhat higher compared with that of the SHHS database. Comparing the validation results of the TVGH independent test set for Model-Saturation trained on 2 different databases in Table 6, it can be seen that using the TVGH database yields better predictions than the SHHS database. This suggests that different predictive models may be needed for different populations.

Finally, the change in feature importance reveals that variance has more significant importance in distinguishing sleep apnea in the TVGH database, implying that the blood oxygen fluctuations in patients with sleep apnea are more notable than those without sleep apnea. Furthermore, the slight increase in frequency from the spectral analysis indicates that the duration of blood oxygen fluctuations is shorter.

### Limitations

There are several limitations that need to be addressed in this study. First, using SpO_2_ signals as a physiological signal for OSA detection is intuitive and widely used because repetitive airway obstruction can cause fluctuations in blood oxygen levels. However, not all respiratory events result in changes in blood oxygen levels. According to the definition of apnea by the AASM, it is based on the degree of airflow reduction, not changes in blood oxygen levels. Therefore, using a single blood oxygen concentration signal may miss such events.

Second, this study used the acceptable definition published by the AASM in 2012 to estimate the AHI. Compared with the recommended definition, the main difference is in the evaluation of hypopnea. According to the acceptable definition, hypopnea is defined as a decrease in airflow of 30% or more for at least 10 seconds, accompanied by a decrease in blood oxygen levels of 4% or more. The recommended definition is defined as a decrease in airflow of 30% or more for at least 10 seconds, accompanied by a decrease in blood oxygen levels of 3% or more or awakening from sleep due to brain wave changes. Due to the differences in definitions, the Model-Saturation may provide relatively lenient results compared to the current standards, resulting in an underestimation of the severity of sleep apnea.

Third, the Model-Saturation was established using the SHHS database, which was collected in the United States and includes middle-aged and elderly participants older than 40 years. Although the advantage of this database is a large amount of home-based polysomnography data, there is still concern about whether the model established from this database is suitable for the Taiwanese population, in terms of social determinants of health and access to health care resources, as well as a generalization to young and middle-aged adults. However, there is currently no similar large-scale home-based polysomnography database in Taiwan for training purposes. If there are future integration efforts for relevant sleep examination data, it may be possible to train models that are more tailored to the needs of Taiwanese patients and more accurate.

Fourth, the Model-Questionnaire trained using the TVGH dataset lacked data from a healthy control group. This dataset consisted exclusively of patients diagnosed with OSA and primary insomnia, which may affect the model’s ability to accurately distinguish healthy participants. Despite this limitation, the PSQI questionnaire, incorporated within our model, served a critical role. In practical clinical settings, it enables the preliminary differentiation between patients and potentially healthy individuals through the identification of nonapnea cases, specifically by using a PSQI score below 5 as a screening criterion.

### Conclusions

This study aimed to develop an efficient and cost-effective sleep apnea screening process by combining 2 machine learning models based on subjective questionnaires and objective SpO_2_ signals. The Model-Questionnaire achieved an *F*_1_-score of 0.86 using key features and the ExtraTrees algorithm, while the Model-Saturation scored 0.85 using CatBoost. Both models demonstrated good predictive ability.

The screening process of combined models started with a questionnaire to identify patients with potential sleep apnea, followed by a SpO_2_ analysis for those classified with sleep apnea. The Model-Saturation showed high precision in classifying moderate to severe sleep apnea cases. This combined approach enhanced screening accuracy and reduced resource waste compared with using solely subjective or objective methods, potentially benefiting practical clinical applications and optimizing resources.

## Abbreviations

AASM: American Academy of Sleep Medicine
AHI: apnea-hypopnea index
AUC: area under curve
MSE: multiscale entropy
ODI: oxygen desaturation index
OSA: obstructive sleep apnea
PSD: power spectral density
PSQI: Pittsburgh Sleep Quality Index
SHHS: Sleep Heart Health Study
SMOTE: Synthesized Minority Oversampling Technique
SpO_2_: blood oxygen saturation
STOP: Snoring, Tiredness, Observed apnea, high Blood Pressure
TVGH: Taipei Veterans General Hospital

## Data Availability

The datasets generated and analyzed during the current study are not publicly available due to a confidentiality agreement required by the institutional review board but are available from the corresponding author on reasonable request.
